# Perceived Neighborhood Environment and Walking for Specific Purposes Among Elderly Japanese

**DOI:** 10.2188/jea.JE20110044

**Published:** 2011-11-05

**Authors:** Shigeru Inoue, Yumiko Ohya, Yuko Odagiri, Tomoko Takamiya, Masamitsu Kamada, Shinpei Okada, Kohichiro Oka, Yoshinori Kitabatake, Tomoki Nakaya, James F Sallis, Teruichi Shimomitsu

**Affiliations:** 1 Department of Preventive Medicine and Public Health, Tokyo Medical University, Tokyo, Japan; 2 Physical Education and Medicine Research Center Unnan, Unnan, Japan; 3 Physical Education and Medicine Research Foundation, Tomi, Japan; 4 Faculty of Sport Sciences, Waseda University, Tokorozawa, Japan; 5 Meiji Yasuda Life Foundation of Health and Welfare, Hachio-ji, Japan; 6 Department of Geography, College of Letters, Ritsumeikan University, Kyoto, Japan; 7 Department of Psychology, San Diego State University, USA

**Keywords:** physical activity, transportation, recreation, policy

## Abstract

**Background:**

Recent research has revealed the importance of neighborhood environment as a determinant of physical activity. However, evidence among elderly adults is limited. This study examined the association between perceived neighborhood environment and walking for specific purposes among Japanese elderly adults.

**Methods:**

This population-based, cross-sectional study enrolled 1921 participants (age: 65–74 years, men: 51.9%). Neighborhood environment (International Physical Activity Questionnaire Environmental Module) and walking for specific purposes (ie, transportation or recreation) were assessed by self-report. Multilevel logistic regression analyses with individuals at level 1 and neighborhoods at level 2 were conducted to examine the association between environment and walking, after adjustment for potential confounders.

**Results:**

Access to exercise facilities, social environment, and aesthetics were associated with total neighborhood walking. Odds ratios (95% CI) were 1.23 (1.00–1.51), 1.39 (1.14–1.71), and 1.48 (1.21–1.81), respectively. Regarding walking for specific purposes, social environment and aesthetics were consistent correlates of both transportation walking and recreational walking. Environmental correlates differed by specific types of walking and by sex. Transportation walking significantly correlated with a greater variety of environmental attributes. Sex differences were observed, especially for transportation walking. Bicycle lanes, crime safety, traffic safety, aesthetics, and household motor vehicles were significant correlates among men, while access to shops, access to exercise facilities, and social environment were important among women.

**Conclusions:**

Specific environment–walking associations differed by walking purpose and sex among elderly adults. Social environment and aesthetics were consistent correlates of both transportation walking and recreational walking. Improving these environmental features might be effective in promoting physical activity among elderly Japanese.

## INTRODUCTION

The health benefits of physical activity have been well documented in previous studies. A physically active lifestyle reduces the risk of all-cause mortality, cardiovascular diseases, diabetes, and some cancers.^1^^–^^4^ In addition, it plays an important role in maintaining functional ability and independence among elderly adults.^5^^–^^8^ However, a large proportion of the population in developed countries remains physically inactive.^9^ In Japan, during the years 1999–2009, mean daily steps decreased from 7962 to 7214 among men and from 7226 to 6352 among women. The trend is similar among elderly Japanese.^10^^,^^11^

To establish effective intervention strategies, evidence of physical activity correlates is needed. Various sociodemographic and psychological factors have been recognized as determinants of physical activity.^12^ In addition, recent studies have identified neighborhood environmental characteristics that are consistently related to physical activity,^13^^–^^19^ including residential density, access to destinations, sidewalks, aesthetics, and access to exercise facilities. Interventions regarding these factors are expected to have a substantial long-term impact on population physical activity levels, which could complement the usually short-term effects of individually targeted interventions.

Studies have revealed that the relationships between environmental factors and physical activity differ with regard to the purpose of physical activity (eg, transportation vs recreational walking), population group (eg, men vs women), and cultural setting (eg, Western vs Eastern).^15^^,^^20^ Age has been shown to be an important modifier of environment–physical activity relationships.^21^ Elderly adults often have different roles in society (eg, retired from work, fewer childcare obligations), different physical activity patterns, and lower fitness levels as compared with young or middle-aged adults. These differences may modify physical activity–environment relationships. Thus, investigation of the relation of physical activity to neighborhood environment among elderly adults is important and of great interest. However, data on elderly adults are not sufficient.^21^^–^^23^ Furthermore, to our knowledge, there has been no published study of these relations among elderly Japanese.

In this study, the associations between various perceived neighborhood environmental attributes and walking for specific purposes were examined in a cross-sectional study using randomly selected community samples of elderly Japanese.

## METHODS

### Participants and data collection

This cross-sectional study was conducted from February through March 2010. A total of 2700 residents aged 65 to 74 years and living in 3 cities in Japan (Bunkyo ward in Tokyo, Fuchu in Tokyo, and Oyama in Shizuoka prefecture) were randomly selected from registries of residential addresses and stratified by sex, age (65–69, 70–74 years), city, and neighborhood. In this study, neighborhood was defined as *cho-cho*, the smallest administrative unit for area in Japan. There were 68 *cho-cho* in Bunkyo, 146 in Fuchu, and 45 in Oyama. First, 15 neighborhoods were randomly selected from each city, and 60 subjects from each neighborhood were randomly selected and stratified by sex and age. As a result, 45 neighborhoods (15 neighborhoods from each city) were selected, and the sample of 2700 older adults included 1350 residents of each sex, 1350 residents of each age category, and 900 subjects from each city. To encompass a large variety of environmental characteristics and walking behaviors, we designed the study to include neighborhoods in urban, suburban, and rural areas. The locations, areas, population sizes, and population densities of each city are shown in the Figure. Bunkyo is in central Tokyo (area: 11.3 km^2^, population: 191 463). Fuchu is a suburban city located about 20 km west of the center of Tokyo (area: 29.3 km^2^, population: 244 834). It is in the Tokyo Metropolitan Area and within commuting distance from central Tokyo. Oyama is a small rural city located about 80 km west of Tokyo (area: 136.1 km^2^, population: 20 783).

**Figure.**
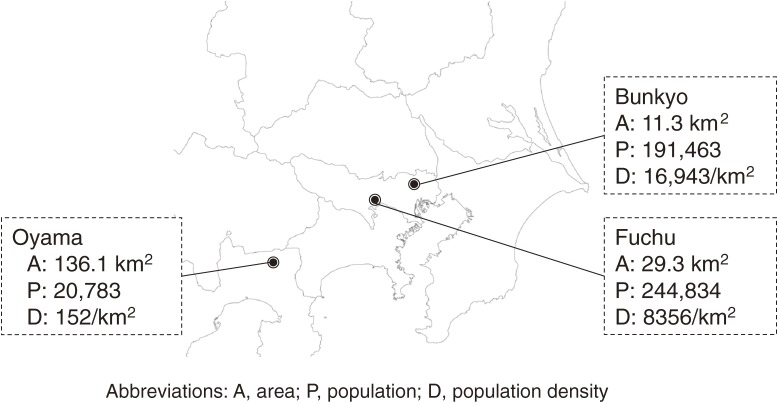
Location and characteristics of the 3 studied cities

For data collection, questionnaires were sent to and collected from participants via post. To obtain a better response rate, invitation letters that described the content of the study were sent to all 2700 subjects 2 weeks before the survey. As an incentive for participation, a 500-yen (about 6 US dollars in 2011) book voucher was offered. During the survey, a call center was set up to respond to survey inquiries from the subjects. Additional requests to complete the survey were mailed twice to nonrespondents. If the survey was incomplete, we asked the participant to redo the survey. As a result, of the 2700 subjects, 2046 responded to the survey. After data cleaning, 1966 subjects had valid data, which were included in the present analyses (response rate: 72.8%). Due to disabilities that prevented walking, the 45 participants who answered, “could not do physical activities,” to the question, “During the past 4 weeks, how much did physical health problems limit your usual physical activities (such as walking or climbing stairs)?,” were excluded from this study. This question was an item from the 8-item Short-Form Health Survey (SF8).^24^ Ultimately, data from 1921 residents were included in the analyses. Participants signed an informed consent document before answering the questionnaire. This study received prior approval from the Tokyo Medical University Ethics Committee.

### Assessment of perceived neighborhood environment

To measure perceived neighborhood environmental characteristics, the International Physical Activity Questionnaire Environmental Module (IPAQ-E) was used.^25^^–^^27^ The survey has demonstrated test-retest reliability^25^ and concurrent validity,^25^^,^^27^ and several items were related to physical activity in an 11-country study.^26^ Questions regarding neighborhood environment attributes were taken or adapted from previous measures developed in the United States.^28^^,^^29^ The IPAQ-E consists of 17 questions: 7 core items, 4 recommended items, and 6 optional items. In this study, we used core and recommended items that assess residential density, access to shops, public transport, sidewalks, bicycle lanes, access to exercise facilities, crime safety, traffic safety, social environment (seeing people being active), aesthetics (the aesthetic and pleasing qualities of a neighborhood for walking), and ownership of household motor vehicles. These questions refer to a neighborhood environment where the person could walk within 10 to 15 minutes from their residence. Nine of 11 items (residential density and household motor vehicles excluded) are statements that describe neighborhood features believed to be related to physical activity, followed by 4 response choices: strongly disagree, somewhat disagree, somewhat agree, and strongly agree. For example, the statement regarding sidewalks is, “There are sidewalks on most of the streets in my neighborhood.” The residential density item asks about the main types of houses in neighborhoods (eg, detached single-family residences, multifamily condos, apartments). The question on motor vehicles concerns the number of motor vehicles in the participant’s household. The translation process, description of each item, and reliability of this scale were reported in a previous study.^25^

### Assessment of neighborhood walking

Participants were asked about their frequency of walking (days/week) and average walking duration each day (min/day) for 5 specific purposes: transportation walking for daily activity, recreational walking, walking for commuting to work, walking during work, and walking for other purposes. A walking questionnaire, whose validity was previously reported,^30^ was modified for this study. The former questionnaire included 6 types of walking, including walking for commuting to school. However, in this study population, few persons were likely to commute to school. Thus, the question on commuting to school was deleted and this type of walking was included in walking for other purposes. The questionnaire instructed participants to report continuous walking done for 5 or more minutes. Walking time (min/week) was calculated as the product of frequency and duration. In this study, we followed the same analytic methods of a previous study^30^ and focused on types of walking that are expected to occur in the participant’s neighborhood. These are (1) transportation walking for daily activity (min/week), (2) recreational walking (min/week), and (3) total neighborhood walking (sum of the time of 3 types of walking: transportation walking for daily activity, recreational walking, and walking for commuting to work, min/week). Although walking for commuting to work is also expected to occur, at least in part, in the neighborhood, we excluded this type of walking from the specific analyses because the present sample included only 331 participants (17.2%) who worked regularly.

### Sociodemographic and other variables

Sex, age, and city of residence were obtained from the registry of residential addresses of each city. Educational attainment (years of education), employment status (working hours), living with family or other cohabitants, and self-rated health were obtained from self-reports. Body mass index (BMI) was calculated from self-reported height and weight. Self-rated health was measured with a single item from SF-8 that asked participants to rate their health. Participants chose the most suitable response from a 6-point scale: excellent, very good, good, fair, poor and very poor, to the question, “Overall, how would you rate your health during the past 4 weeks?”.^24^

### Statistical analyses

To examine the association between neighborhood environment as the independent variable and walking as the dependent variable, odds ratios (ORs) and 95% CIs for active walkers were calculated using multilevel logistic regression models with individuals at level 1 and neighborhood at level 2. In other words, we used a generalized linear mixed model with each environmental variable and control covariate as fixed effects, with a random intercept term at the neighborhood level. City of residence was included in the model as a dummy variable. This approach was chosen to account for the nested data structure. For the analyses, responses to 11 environmental variables were converted into dichotomous variables by means of a method used in previous studies.^25^^,^^26^^,^^31^ For residential density, the choice of “detached single-family residences” formed a category indicating low residential density, while others were included in another category, indicating high residential density. As for the number of household motor vehicles, responses were categorized as none and 1 or more. For the other 9 environmental variables, responses were classified into 2 categories: agreement (strongly agree and somewhat agree) and disagreement (somewhat disagree and strongly disagree). For walking variables, participants were classified into 2 groups. First, we analyzed relationships between total neighborhood walking and environmental variables. Participants were classified as an active neighborhood walker if they walked 150 minutes/week or more, consistent with current physical activity guidelines.^5^ Then, 2 specific types of walking—transportation walking for daily activity and recreational walking—were analyzed. For these variables, participants were divided into 2 groups by using the median: less than 60 minutes or 60 minutes or more per week for transportation walking for daily activity and less than 30 or 30 minutes or more per week for recreational walking. To calculate ORs, the references were set as environmental factors expected to be associated with lower levels of walking (low for residential density, owning household motor vehicles, and poor for the other 9 variables), meaning that an OR greater than 1.00 indicates an expected association between an activity-supportive environmental characteristic and active walking. ORs were adjusted by age, sex, employment status (working for 35+ hours per week vs less, including no work), educational level (13+ years vs <13 years), BMI (25+ kg/m^2^ vs <25 kg/m^2^), and self-rated health (good: excellent, very good, good vs fair or poor: fair, poor, very poor). A *P* value less than 0.05 was considered to indicate statistical significance. Analyses were conducted with IBM SPSS Statistics version 19 (SPSS Inc., Tokyo, Japan), for descriptive analyses, and lme4 (ver. 0.999375-39), a mixed modeling package for R (ver. 2.12.1), which is a free software environment for statistical computing for multilevel analyses.

## RESULTS

Table 1 shows the characteristics of the participants. Men accounted for 50.9% of this sample. The mean age (SD) was 69.5 (2.9) years. The city of residence was Bunkyo for 31.4% of participants, Fuchu for 34.3%, and Oyama for 34.3%. Overall, 17.2% of participants worked 35 hours/week or longer, and 10.6% were living alone. As for walking time, the median time (25th percentile, 75th percentile) was 165 (45, 350) minutes/week for total neighborhood walking, 60 (0, 140) minutes/week for transportation walking for daily activity, and 30 (0, 180) minutes/week for recreational walking.

**Table 1.** Characteristics of participants

	Overall *n* = 1921		Men *n* = 977		Women *n* = 944	
			
	*n*	%	*n*	%	*n*	%
Age, years						
65–69	956	49.8	488	49.9	468	49.6
70–74	965	50.2	489	50.1	476	50.4
mean ± SD	69.5 ± 2.9		69.5 ± 3.0		69.6 ± 2.9	
City of residence						
Bunkyo	604	31.4	311	31.8	293	31.0
Fuchu	658	34.3	335	34.3	323	34.2
Oyama	659	34.3	331	33.9	328	34.7
Education, years						
<13	1254	65.3	565	57.8	689	73.0
13+	667	34.7	412	42.2	255	27.0
Employment status						
35+ hours/week	331	17.2	248	25.4	83	8.8
no work ​ or <35 hours/week	1590	82.8	729	74.6	861	91.2
Living with family or other cohabitants						
Yes	1718	89.4	888	90.9	830	87.9
No	203	10.6	89	9.1	114	12.1
BMI, kg/m^2^						
<25	1526	79.4	751	76.9	775	82.1
25+	395	20.6	226	23.1	169	17.9
Mean ± SD	22.8 ± 3.0		23.2 ± 2.7		22.3 ± 3.3	
Self-rated health						
Excellent	57	3.0	31	3.2	26	2.8
Very good	432	22.5	231	23.6	201	21.3
Good	1092	56.8	559	57.2	533	56.5
Fair	265	13.8	120	12.3	145	15.4
Poor	59	3.1	26	2.7	33	3.5
Very poor	16	0.8	10	1.0	6	0.6
Total neighborhood walking, min/week						
<150	874	45.8	442	45.5	432	46.1
150+	1035	54.2	530	54.5	505	53.9
Median ​ (25%tile, 75%tile)	165 (45, 350)		178 (45, 370)		160 (47, 320)	
Transportation walking for daily activity, min/week						
<60	945	49.4	560	57.5	385	41.0
60+	967	50.6	414	42.5	553	59.0
Median ​ (25%tile, 75%tile)	60 (0, 140)		30 (0, 120)		80 (0, 160)	
Recreation walking, min/week						
<30	921	48.0	421	43.1	500	53.0
30+	999	52.0	555	56.9	444	47.0
Median ​ (25%tile, 75%tile)	30 (0, 180)		60 (0, 200)		5 (0, 120)	

Abbreviation: BMI, body mass index.

Table 2 shows the results of analyses of total neighborhood walking. Access to exercise facilities (OR 1.23, 95% CI 1.00–1.51), social environment (1.39, 1.14–1.71), and aesthetics (1.48, 1.21–1.81) were significantly associated with total neighborhood walking. Aesthetics was a consistent correlate of total walking among men and women. Social environment was related to total walking only among men, and access to shops was significant only among women.

**Table 2.** Odds ratios of active neighborhood walkers^a^ by environmental factors

	Overall				Men				Women			
			
	% active walkers^e^	OR^b,d^	95% CI	*P* value	% active walkers^e^	OR^c,d^	95% CI	*P* value	% active walkers^e^	OR^c,d^	95% CI	*P* value
Residential density												
High	59.7 (427/715)	1.01	0.81–1.25	0.937	57.9 (210/363)	0.93	0.69–1.26	0.650	61.6 (217/352)	1.09	0.80–1.49	0.578
Low	51.0 (606/1189)				52.7 (320/607)				49.1 (286/582)			
Access to shops												
Good	57.8 (747/1292)	1.23	0.99–1.52	0.061	57.1 (385/674)	1.02	0.75–1.39	0.913	58.6 (362/618)	1.44	1.07–1.94	0.017
Poor	46.7 (281/602)				48.6 (142/292)				44.8 (139/310)			
Public transport												
Good	55.0 (955/1735)	0.99	0.71–1.40	0.976	55.1 (482/875)	0.94	0.59–1.49	0.799	55.0 (473/860)	1.08	0.65–1.80	0.776
Poor	46.4 (77/166)				48.4 (45/93)				43.8 (32/73)			
Sidewalks												
Good	56.4 (868/1540)	1.26	0.98–1.61	0.067	56.2 (438/779)	1.22	0.87–1.72	0.255	56.5 (430/761)	1.30	0.91–1.85	0.152
Poor	45.4 (163/359)				47.6 (89/187)				43.0 (74/172)			
Bicycle lanes												
Good	58.1 (403/694)	1.08	0.88–1.32	0.480	56.7 (200/353)	0.98	0.74–1.31	0.904	59.5 (203/341)	1.18	0.88–1.57	0.271
Poor	52.2 (628/1204)				53.3 (327/613)				50.9 (301/591)			
Access to exercise facilities												
Good	57.2 (700/1224)	1.23	1.00–1.51	0.047	56.9 (354/622)	1.19	0.90–1.59	0.221	57.5 (346/602)	1.31	0.98–1.76	0.072
Poor	49.2 (332/675)				50.3 (174/346)				48.0 (158/329)			
Crime safety												
Good	55.1 (717/1302)	0.96	0.79–1.18	0.715	54.0 (388/719)	0.78	0.57–1.05	0.106	56.4 (329/583)	1.16	0.87–1.53	0.308
Poor	52.8 (315/597)				56.4 (141/250)				50.1 (174/347)			
Traffic safety												
Good	56.1 (715/1275)	1.15	0.94–1.40	0.168	55.5 (372/670)	1.08	0.81–1.43	0.606	56.7 (343/605)	1.24	0.94–1.65	0.130
Poor	51.1 (318/622)				52.5 (156/297)				49.8 (162/325)			
Social environment												
Good	57.4 (752/1309)	1.39	1.14–1.71	0.001	58.3 (395/677)	1.57	1.18–2.09	0.002	56.5 (357/632)	1.24	0.93–1.66	0.145
Poor	47.7 (279/585)				46.2 (134/290)				49.2 (145/295)			
Aesthetics												
Good	57.2 (743/1299)	1.48	1.21–1.81	<0.001	58.1 (376/647)	1.56	1.18–2.07	0.002	56.3 (367/652)	1.38	1.03–1.85	0.030
Poor	48.2 (290/602)				47.7 (153/321)				48.8 (137/281)			
Household motor vehicles												
0	64.0 (326/509)	1.21	0.95–1.55	0.126	63.1 (140/222)	1.19	0.84–1.69	0.324	64.8 (186/287)	1.18	0.83–1.66	0.361
1+	50.8 (706/1391)				52.1 (390/749)				49.2 (316/642)			

Abbreviations: OR, odds ratio; CI, confidence interval; BMI, body mass index.

^a^An active neighborhood walker was defined as total neighborhood walking of 150+ min/week.

^b^Odds ratios were adjusted for age, sex, city of residence, employment status, educational level, BMI, and self-rated health.

^c^Odds ratios were adjusted for age, city of residence, employment status, educational level, BMI, and self-rated health.

^d^Reference is environmental characteristics expected to be associated with a lower level of physical activity, ie, an odds ratio greater than 1.00 indicates an expected positive association.

^e^Figures in parentheses are number of active walkers/number of participants in the category.

Results of analyses of the 2 specific purposes of walking are shown in Tables 3 and 4. Among the overall sample, 2 environmental attributes—social environment and aesthetics—were consistent correlates of these 2 types of walking. In addition, bicycle lanes, access to exercise facilities, and household motor vehicles were significantly associated with transportation walking. Sex differences were observed, especially in the analyses of transportation walking. Among men, significant correlates of this type of walking were bicycle lanes, crime safety, traffic safety, aesthetics, and household motor vehicles. Among women, access to shops, access to exercise facilities, and social environment were related to this type of walking. The ORs for the majority of significant relationships were greater than 1.00, ie, in the expected direction of correlations, except crime safety and traffic safety in the analyses of transportation walking among men. Most variance estimates of error terms at level 2 were close to 0, so estimated coefficients of fixed effects were almost identical to those in conventional single-level logistic regression.

**Table 3.** Odds ratios of active transportation walkers^a^ by environmental factors

	Overall				Men				Women			
			
	% active walkers^e^	OR^b,d^	95% CI	*P* value	% active walkers^e^	OR^c,d^	95% CI	*P* value	% active walkers^e^	OR^c,d^	95% CI	*P* value
Residential density												
High	56.8 (407/717)	1.03	0.83–1.28	0.815	48.1 (175/364)	1.17	0.87–1.58	0.299	65.7 (232/353)	0.87	0.62–1.20	0.395
Low	47.0 (559/1190)				39.3 (239/608)				55.0 (320/582)			
Access to shops												
Good	54.1 (701/1295)	1.22	0.98–1.51	0.076	44.1 (298/676)	0.95	0.69–1.30	0.747	65.1 (403/619)	1.57	1.16–2.13	0.004
Poor	43.5 (262/602)				39.0 (114/292)				47.7 (148/310)			
Public transport												
Good	51.6 (896/1738)	1.03	0.73–1.46	0.856	42.9 (376/877)	0.98	0.61–1.57	0.933	60.4 (520/861)	1.15	0.67–1.96	0.607
Poor	41.0 (68/166)				38.7 (36/93)				43.8 (32/73)			
Sidewalks												
Good	52.6 (811/1543)	1.19	0.92–1.53	0.180	43.8 (342/781)	1.20	0.84–1.70	0.317	61.5 (469/762)	1.20	0.83–1.73	0.333
Poor	42.3 (152/359)				37.4 (70/187)				47.7 (82/172)			
Bicycle lanes												
Good	56.8 (395/695)	1.26	1.03–1.54	0.026	49.2 (174/354)	1.41	1.06–1.87	0.019	64.8 (221/341)	1.14	0.85–1.54	0.385
Poor	47.2 (569/1206)				38.8 (238/614)				55.9 (331/592)			
Access to exercise facilities												
Good	53.7 (658/1225)	1.26	1.03–1.55	0.027	44.3 (276/623)	1.16	0.87–1.54	0.319	63.5 (382/602)	1.39	1.03–1.88	0.033
Poor	45.1 (305/677)				39.5 (137/347)				50.9 (168/330)			
Crime safety												
Good	49.9 (650/1302)	0.88	0.72–1.09	0.240	41.0 (295/719)	0.69	0.51–0.94	0.017	60.9 (355/583)	1.07	0.80–1.42	0.665
Poor	52.3 (314/600)				47.2 (119/252)				56.0 (195/348)			
Traffic safety												
Good	50.0 (637/1275)	0.90	0.74–1.10	0.321	40.1 (269/670)	0.71	0.53–0.94	0.017	60.8 (368/605)	1.15	0.86–1.54	0.353
Poor	52.3 (327/625)				48.2 (144/299)				56.1 (183/326)			
Social environment												
Good	53.0 (695/1311)	1.31	1.06–1.61	0.011	44.1 (299/678)	1.20	0.90–1.60	0.223	62.6 (396/633)	1.42	1.06–1.92	0.021
Poor	45.7 (268/586)				39.5 (115/291)				51.9 (153/295)			
Aesthetics												
Good	52.7 (685/1301)	1.31	1.07–1.61	0.009	44.8 (290/648)	1.33	1.00–1.76	0.047	60.5 (395/653)	1.28	0.95–1.74	0.107
Poor	46.4 (280/603)				38.5 (124/322)				55.5 (156/281)			
Household motor vehicles												
0	64.7 (330/510)	1.43	1.12–1.83	0.004	55.4 (123/222)	1.54	1.10–2.17	0.012	71.9 (207/288)	1.32	0.92–1.92	0.135
1+	45.4 (633/1393)				38.7 (291/751)				53.3 (342/642)			

Abbreviations: OR, odds ratio; CI, confidence interval; BMI, body mass index.

^a^An active transportation walker was defined as transportation walking for a daily activity of 60+ min/week.

^b^Odds ratios were adjusted for age, sex, city of residence, employment status, educational level, BMI, and self-rated health.

^c^Odds ratios were adjusted for age, city of residence, employment status, educational level, BMI, and self-rated health.

^d^Reference is environmental characteristics expected to be associated with lower levels of physical activity, ie, an odds ratios greater than 1.00 indicates an expected positive association.

^e^Figures in parentheses are number of active walkers/number of participants in the category.

**Table 4.** Odds ratios of active recreational walkers^a^ by environmental factors

	Overall				Men				Women			
			
	% active walkers^e^	OR^b,d^	95% CI	*P* value	% active walkers^e^	OR^c,d^	95% CI	*P* value	% active walkers^e^	OR^c,d^	95% CI	*P* value
Residential density												
High	54.6 (393/720)	1.04	0.84–1.30	0.698	57.3 (209/365)	0.89	0.65–1.22	0.472	51.8 (184/355)	1.23	0.90–1.67	0.187
Low	50.6 (605/1195)				56.8 (346/609)				44.2 (259/586)			
Access to shops												
Good	54.4 (707/1300)	1.22	0.99–1.51	0.066	59.4 (402/677)	1.12	0.82–1.54	0.481	49.0 (305/623)	1.30	0.97–1.75	0.080
Poor	47.3 (286/605)				51.5 (151/293)				43.3 (135/312)			
Public transport												
Good	52.1 (909/1744)	0.98	0.69–1.38	0.896	57.2 (502/878)	0.96	0.60–1.53	0.867	47.0 (407/866)	0.99	0.60–1.64	0.965
Poor	50.6 (85/168)				54.3 (51/94)				45.9 (34/74)			
Sidewalks												
Good	52.8 (818/1550)	1.09	0.85–1.40	0.500	57.2 (448/783)	0.98	0.69–1.39	0.915	48.2 (370/767)	1.20	0.84–1.71	0.310
Poor	49.7 (179/360)				56.7 (106/187)				42.2 (73/173)			
Bicycle lanes												
Good	51.8 (362/699)	0.94	0.77–1.15	0.567	54.4 (193/355)	0.77	0.58–1.04	0.088	49.1 (169/344)	1.11	0.84–1.48	0.456
Poor	52.2 (632/1210)				58.4 (359/615)				45.9 (273/595)			
Access to exercise facilities												
Good	53.5 (659/1232)	1.17	0.95–1.43	0.132	57.8 (362/626)	1.10	0.83–1.48	0.502	49.0 (297/606)	1.27	0.95–1.70	0.104
Poor	49.7 (337/678)				55.2 (191/346)				44.0 (146/332)			
Crime safety												
Good	52.9 (691/1307)	0.97	0.79–1.19	0.751	57.6 (415/721)	0.97	0.72–1.32	0.862	47.1 (276/586)	0.95	0.72–1.25	0.715
Poor	50.2 (303/603)				55.2 (139/252)				46.7 (164/351)			
Traffic safety												
Good	53.2 (680/1278)	1.07	0.87–1.30	0.530	56.9 (382/671)	0.93	0.70–1.24	0.633	49.1 (298/607)	1.20	0.91–1.59	0.202
Poor	50.0 (315/630)				57 (171/300)				43.6 (144/330)			
Social environment												
Good	55.2 (727/1318)	1.42	1.16–1.75	0.001	61.2 (416/680)	1.73	1.29–2.31	<0.001	48.7 (311/638)	1.18	0.89–1.58	0.249
Poor	45.7 (268/587)				47.4 (138/291)				43.9 (130/296)			
Aesthetics												
Good	55.2 (722/1308)	1.55	1.26–1.89	<0.001	60.5 (394/651)	1.59	1.20–2.11	0.001	49.9 (328/657)	1.47	1.10–1.97	0.009
Poor	45.2 (273/604)				49.2 (158/321)				40.6 (115/283)			
Household motor vehicles												
0	55.8 (285/511)	1.01	0.79–1.29	0.937	59.6 (133/223)	0.92	0.65–1.31	0.655	52.8 (152/288)	1.07	0.76–1.50	0.705
1+	50.7 (710/1400)				56.1 (422/752)				44.4 (288/648)			

Abbreviations: OR, odds ratio; CI, confidence interval; BMI, body mass index.

^a^An active walker was defined as recreational walking 30+ min/week.

^b^Odds ratios were adjusted for age, sex, city of residence, employment status, educational level, BMI, and self-rated health.

^c^Odds ratios were adjusted for age, city of residence, employment status, educational level, BMI, and self-rated health.

^d^Reference is environmental characteristics expected to be associated with lower levels of physical activity, ie, an odds ratios greater than 1.00 indicates an expected positive association.

^e^Figures in parentheses are number of active walkers/number of participants in the category.

## DISCUSSION

This cross-sectional study revealed that neighborhood environmental characteristics were associated with walking, and that these associations varied by purpose, among elderly Japanese. Social environment (viewing people being active) and aesthetics were consistently associated with transportation walking and recreational walking. In addition, among the overall sample, transportation walking was related to 3 other environmental characteristics: bike lanes, access to exercise facilities, and household motor vehicles.

A position statement by the Heart Foundation’s National Physical Activity Advisory Committee in Australia summarized recent studies of neighborhood environmental characteristics and walking in adults.^32^ The statement highlighted 4 characteristics that were consistent correlates of transportation walking—population density, proximity of destinations (including shops and public transport), mixed land-use planning, and street connectivity (the “walkability index” often comprises these attributes)—as well as 3 characteristics that were correlates of recreational walking—access to exercise facilities, pedestrian infrastructure, and aesthetics. The results of our previous study of a Japanese adult sample were consistent with the Committee’s statement.^30^ However, the results of the present study of a sample of elderly adults slightly differed from these previous findings. Residential density and access to public transport have been frequently reported as correlates of transportation walking, but they were not associated with any type of walking in the present study. Access to shops—a consistent and strong correlate of transportation walking in previous studies of adults—was significantly associated with transportation walking only among women. Thus, the present results suggest a relatively weaker association between transportation walking and “walkability”-related environmental features, especially among older men, as compared with young and middle-aged populations. On the other hand, sidewalks, access to exercise facilities, and aesthetics, which were previously reported as correlates of recreational walking, were significantly associated with transportation walking in the present study. These results indicate that transportation walking among elderly adults might have similar characteristics to recreational walking. One possibility is that, as Shigematsu et al^21^ discussed in a previous report, elderly adults might combine transportation walking with recreational walking in 1 trip. For example, someone goes to a park for a recreational walk and, on the way home, goes shopping. Thus, the differentiation between the 2 types of walking may be less clear among elderly adults. Another possibility is that elderly people have fewer obligations than do younger people with regard to daily activities such as commuting and shopping; thus, transportation walking is not a requirement for them. They might walk for transport only when they live in enjoyable and attractive environments that support recreational walking among younger adults.

Before the present analyses, we speculated that elderly people may be vulnerable to unfavorable environments, meaning that the association between environment and physical activity might be stronger among this population. Contrary to expectations, the odds ratios were relatively low in this study, although there is no criterion with which to assess strong versus weak associations. A previous study that examined age differences in the relationship of perceived neighborhood environment to walking reported somewhat similar results, ie, there were a smaller number of environmental features that were significantly associated with walking among older adults as compared with younger adults.^21^ One possible explanation for different and weaker associations among elderly adults is that many elderly people have multiple physical and social limitations, which can lead to complicated associations between environment and physical activity and thus weaken observed associations. For example, patterns of associations may differ by physical fitness level, having or not having a social role, the presence of family members, etc. Further studies are needed to examine if elderly people are vulnerable to neighborhood environmental attributes and if physical and social conditions modify these relationships.

Sex differences were observed in the present study, especially in transportation walking for daily activity. Among men, environmental attributes that were significantly associated with walking for daily activity were bike lanes, traffic safety, crime safety, aesthetics, and household motor vehicles; among women, the associated attributes were access to shops, access to exercise facilities, and social environment. Patterns of associations in women were similar to those noted in Western studies, ie, access to shops and exercise facilities were important for transportation walking.^21^^,^^23^ It is plausible that access to shops is more important for women, who often have more obligations with regard to household duties. Access to exercise facilities was associated with transportation walking but not with recreational walking. This may seem surprising, but was also reported in a previous study.^21^ It may be that adults walk for transport to exercise facilities, take a child to the facilities, including parks, and combine transportation walking and recreational walking. Regarding older men, the relationship between household motor vehicle access and transportation walking suggests a car-dependent lifestyle among this population: those with cars walked less. We observed unexpected negative associations of crime safety and traffic safety with transportation walking among men. We hypothesized that safety was an important issue among elderly adults. However, considering these results, the young old (age 65–74 years) may be sufficiently physically fit and not overly concerned by safety issues. Also, variation in crime safety in Japan might be too low to yield associations, as the country is generally considered safe. Instead, walkers perceived more safety concerns than did sedentary persons. Perhaps walkers are more likely to encounter threats or are more familiar with signs of disorder in their neighborhoods. This is a limitation of a perceived measure and the present cross-sectional design. Because the results of previous studies of safety concerns were inconsistent,^22^^,^^33^^,^^34^ further studies of elderly samples are needed.

There are several limitations of this study. First, this study had a cross-sectional design, which does not allow us to address the direction of causality. Longitudinal or intervention studies are needed in future research. Second, both environmental and walking measures were based on self-reporting. Although self-reporting can assess walking for different purposes and a wide range of environmental characteristics, we must consider the possibility of a discrepancy between perception and reality, even though the measures have been validated. Thus, studies using objective measures, such as accelerometers for physical activity and a geographic information system for environmental evaluation, are needed in the future, although both objective and perceived measures are useful. Third, this study examined elderly adults aged 65 to 74 years. The results could differ among older age groups. We must be careful to consider the generalizability of the results.

Despite these limitations, studies of the physical activity environment for elderly adults are limited, especially among Japanese populations. The present results indicate that several environmental factors are related to walking for specific purposes among elderly Japanese. Improving these environmental features in neighborhoods might be an effective strategy to promote physical activity among this population.
